# Negative soil moisture-precipitation feedback in dry and wet regions

**DOI:** 10.1038/s41598-018-22394-7

**Published:** 2018-03-05

**Authors:** Lingbin Yang, Guoqing Sun, Lu Zhi, Jianjun Zhao

**Affiliations:** 1 Northeast Normal University, School of Geographical Sciences, Changchun, Jilin, 130024 China; 2 Central South University, School of Geosciences and Info-Physics, Changsha, Hunan 410083 China; 3 Information Engineering University, Institute of Geography and Space Information, Zhengzhou, Henan 450001 China

## Abstract

Soil moisture-precipitation (SM-P) feedback significantly influences the terrestrial water and energy cycles. However, the sign of the feedback and the associated physical mechanism have been debated, leaving a research gap regarding global water and climate changes. Based on Koster’s framework, we estimate SM-P feedback using satellite remote sensing and ground observation data sets. Methodologically, the sign of the feedback is identified by the correlation between monthly soil moisture and next-month precipitation. The physical mechanism is investigated through coupling precipitation and soil moisture (P-SM), soil moisture ad evapotranspiration (SM-E) and evapotranspiration and precipitation (E-P) correlations. Our results demonstrate that although positive SM-P feedback is predominant over land, non-negligible negative feedback occurs in dry and wet regions. Specifically, 43.75% and 40.16% of the negative feedback occurs in the arid and humid climate zones. Physically, negative SM-P feedback depends on the SM-E correlation. In dry regions, evapotranspiration change is soil moisture limited. In wet regions, evapotranspiration change is energy limited. We conclude that the complex SM-E correlation results in negative SM-P feedback in dry and wet regions, and the cause varies based on the environmental and climatic conditions.

## Introduction

Soil moisture is a critical state variable in Earth systems and plays a significant role in land-atmosphere interactions^1–3^. The spatial and temporal patterns of soil moisture depend on the variability of precipitation, evapotranspiration and runoff^4,5^. Soil moisture is also a feedback mechanism for other hydrological variables^6–8^. The interactions between soil moisture and the hydrological variables are essential for understanding the physical mechanisms of global water and climate change. Therefore, clarification of the interactions has become a hotspot in global change studies. However, the complexity of the interactions and the scarcity of observations available to characterize the relevant processes have become increasingly apparent, resulting in considerable uncertainty in related studies^9–11^.

Soil moisture-precipitation (SM-P) feedback is a central issue in studies of land-atmosphere interactions. It has the potential to propagate hydrological conditions and improve the predictability of the land-atmosphere system^12^. Identifying such regions of strong coupling is critical for improving the accuracy of climate prediction. It is also important for water monitoring in regions with strong SM-P coupling^13^. Due to the complex interactions between other hydrological and climatic factors, SM-P feedback varies in signal and strength. Most previous studies found positive correlations between soil moisture and precipitation^14–16^. This process can be interpreted by the water and energy cycles. Based on the water balance, wet soil results in high evapotranspiration, which provides abundant vapor for forming precipitation^11^. From the viewpoint of an energy balance, wet soil enhances the net solar and terrestrial radiation by the decreasing surface albedo and Bowen ratio, leading to a larger total flux of heat from the surface to the boundary layer. These effects then increase moist static energy, local convective storms, and, ultimately, precipitation^16^. However, the opposite phenomenon has been observed in recent studies, which observed that more precipitation fell over dry soil due to the enhanced convective system^17,18^. This phenomenon was further confirmed by studies of global water change, which revealed an overestimation trend based on the paradigm of “dry gets drier, wet gets wetter”^19,20^. The studies were controversial and generated considerable uncertainty regarding SM-P feedback.

Several reasons account for the complexity of SM-P feedback. Most researchers attribute the complexity to the variability associated with different temporal^12^ and spatial scales^21^. Hohenegger *et al*.^22^ observed positive feedback in a 25-km simulation and predominantly negative feedback in a 2.2-km simulation. Ford *et al*.^23,24^ observed positive and negative feedbacks simultaneously in Oklahoma during 19 temporal events, and the probability of afternoon precipitation over wet or dry soils depended on the absence of the low-level jet. Guillod *et al*.^18^ noted that precipitation is more likely to occur in wetter conditions temporally and drier conditions spatially. Recently, several researchers noted that both positive and negative feedbacks exist, even at the same spatial and temporal scales. For example, Findell and Eltahir^25^ identified positive feedbacks in much of the eastern United States and negative feedbacks in the arid southwest, which has a dryline and monsoon season. Tuttle and Salvucci^26^ also found positive and negative feedbacks across the United Sates, although their patterns were different compared to the results of Findell and Eltahir^25^. The results suggest that in addition to the spatial and temporal scales, other factors affect the sign of SM-P feedback. However, the physical mechanism remains unclear and requires further investigation.

The introduction above suggests that SM-P feedback is crucial in land-atmosphere interactions. However, there is significant debate regarding the sign of the feedback and the physical mechanism that controls it, leaving a large gap in climate and hydrology research. This study aims to identify the feedback and evaluate the cause. Section 2 describes the patterns of the SM-P feedback and discusses the physical mechanism. Section 3 provides conclusions and discussion. Section 4 presents the study materials and methods. The study improves the understanding of the physical mechanism of SM-P feedback and supports the effective management of water resources in the context of climatic and anthropogenic change.

## Results

**Figure 1:**
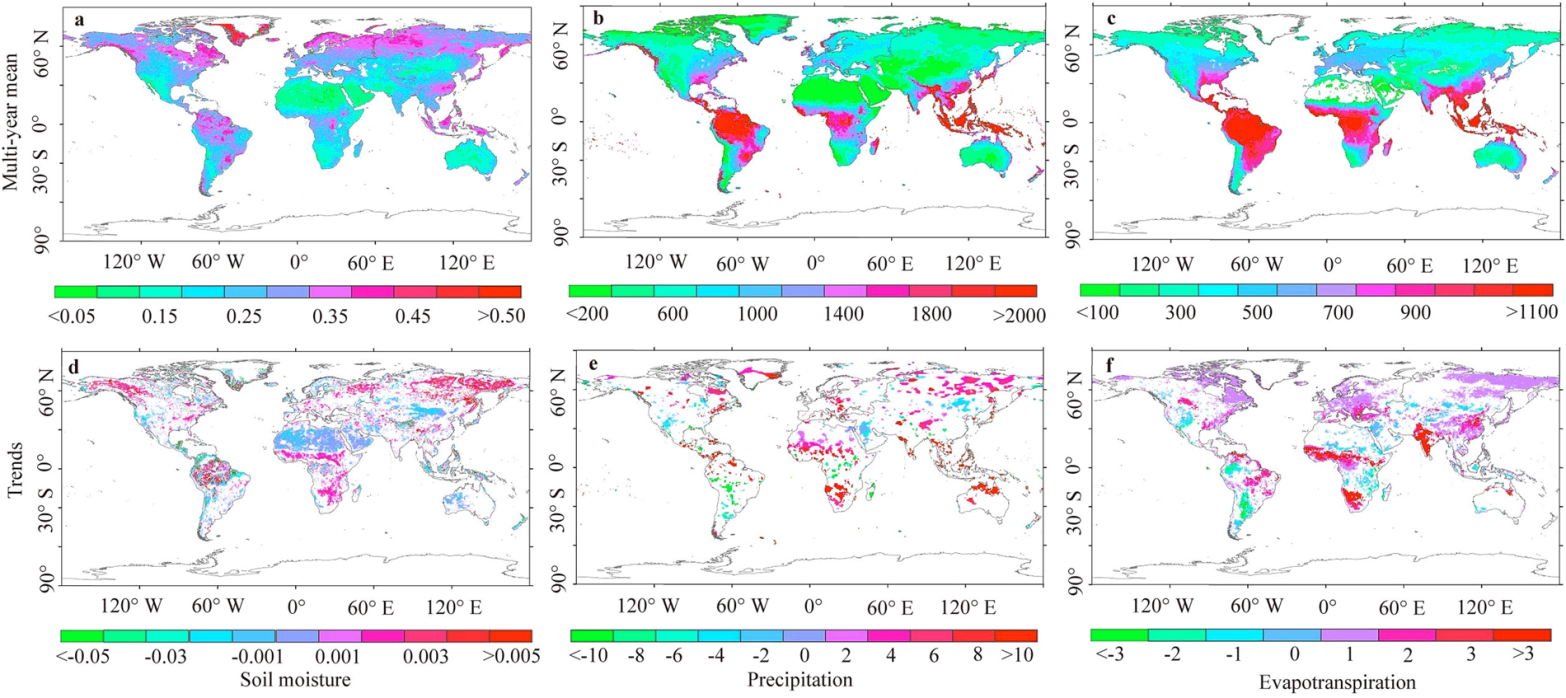
The spatial patterns and trends of soil moisture ((**a**) cm^3^/cm^3^ and (**d**) cm^3^/cm^3^ per year), precipitation ((**b**) mm and (**e**) mm per year) and evapotranspiration ((**c**) mm and (**f**) mm per year) from 1982 to 2011(Created by ArcGIS, version 10.1, http://www.esri.com/).

Overall trends of global water change. In the past 30 years (1982–2011), the global water balance has changed profoundly due to natural and anthropogenic influences. Figure 1(a,d) show the spatial pattern and trend of global soil moisture, with a multi-year mean of 0.226 cm^3^/cm^3^. Dry soils are primarily located in northern Africa, southwestern Europe, central Asia and Australia. Wet areas are mainly located in northern South America, northern Europe and southeastern Asia. Temporally, the global soil moisture increased slightly based on the relationship *y* = 0.0006*x* +0.2174 (*R*^2^ = 0.21, *p* < 0.001; where *y* and *x* represent the soil moisture and calendar year). Specifically, approximately 19.49% of land became drier by −0.0015 cm^3^/cm^3^ per year, and 16.80% became wetter by 0.0021 cm^3^/cm^3^ per year. With respect to precipitation (Fig. 1(b,e)), the multi-year mean is 711.45 mm, which decreases from the equator to the poles spatially. Meanwhile, temporal global precipitation exhibited a significant increase over the past 30 year (*y* = 0.7672*x* +699.56, *R*^2^ = 0.28, *p* < 0.001). Specifically, it increased over 11.17% of the total land area by 7.38 mm per year and decreased over 4.65% of the total land area by −7.16 mm per year. The pattern and trend of evapotranspiration can be observed in Fig. 1(c,f). The multi-year mean of global evapotranspiration is 476.93 mm, with a temporal trend of *y* = 0.2812*x* +472.57 (*R*^2^ = 0.31, *p* < 0.001). It increased in 26.08% of the total land area by 1.10 mm per year and decreased in 6.87% of the total land area by −1.28 mm per year. As shown in Fig. 1(c,e), the spatial pattern of evapotranspiration is highly consistent with that of precipitation, suggesting that a strong positive correlation exists between the two variables.

**Figure 2:**
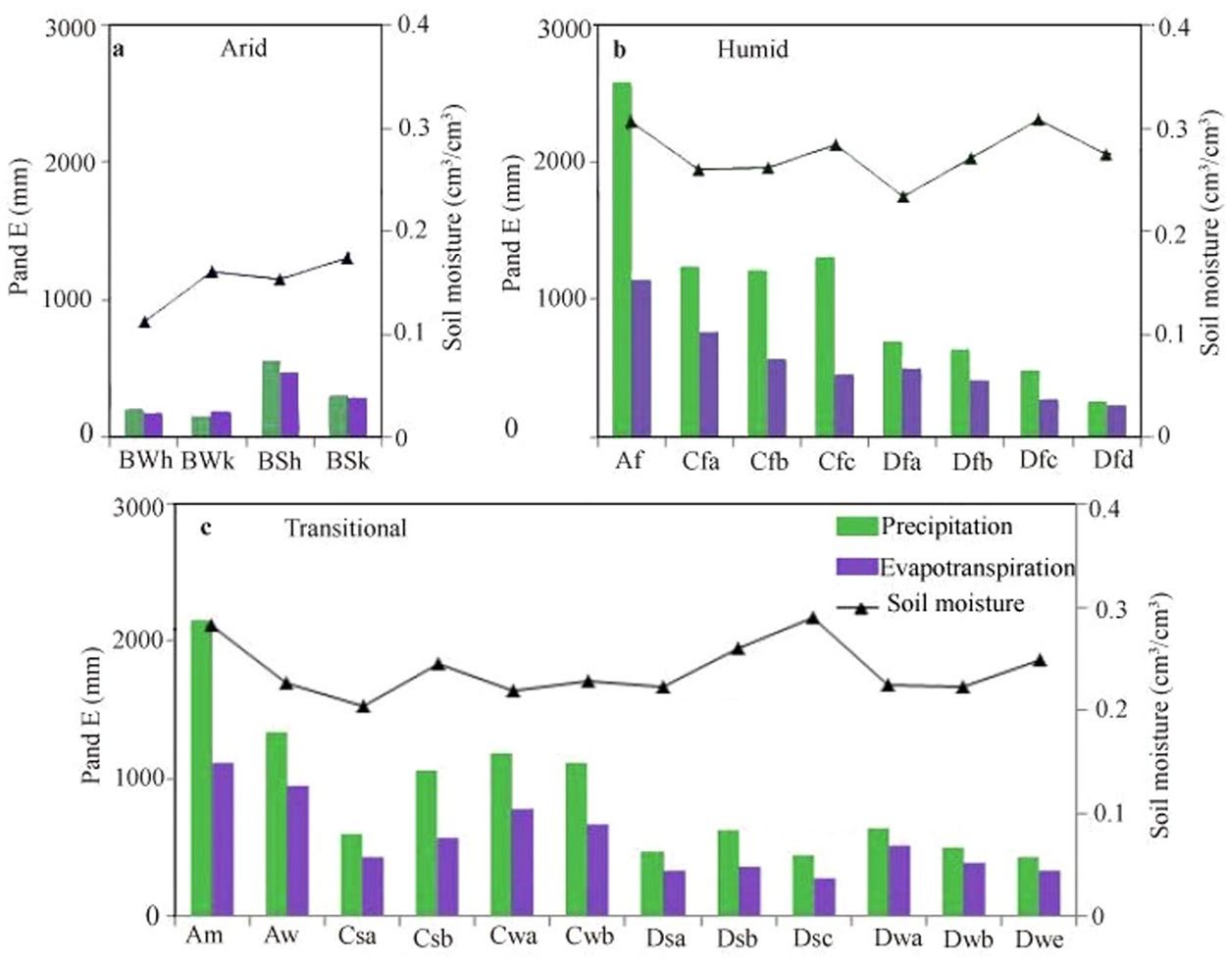
Soil moisture, precipitation and evapotranspiration in different climate zones. The first letter (A to D) refers to the broad climate type: tropical, arid, temperate or cold. The second letter (f, m, w/W and s/S) represents the subsequent precipitation conditions. The third letter (**a**–**d**,**h** and **k**) refers to the temperature classification. The definitions of the climate zones can be found in Peel *et al*.^46^.

Climate is one of the dominant factors that affects global water change. Therefore, we analyze the spatial variability in soil moisture, precipitation and evapotranspiration at the climate zone scale. Thus, the hydrological responses to different climate conditions can be easily estimated. As shown in Fig. 2, soil moisture and precipitation are highest in the humid zone (0.275 cm^3^/cm^3^ and 1057.60 mm), followed by the transitional zone (0.240 cm^3^/cm^3^ and 882.64 mm) and arid zone (0.150 cm^3^/cm^3^ and 306.94 mm). Although precipitation and soil moisture are high in the humid zone, the corresponding evapotranspiration (550.22 mm) is lower than that in the transitional zone (562.85 mm). This inconsistency suggests that complex interactions exist between the three variables. Further analysis shows that the ratio of evapotranspiration to precipitation (E/P) is 0.98, 0.59 and 0.67 in arid, humid and transitional zones, respectively. This result implies that almost all precipitation in arid zones returns to the atmosphere via evaporation from the soil. However, in humid zones, a limited portion of the water evaporates, even from wet soil.

### General pattern of SM-P feedback

**Figure 3:**
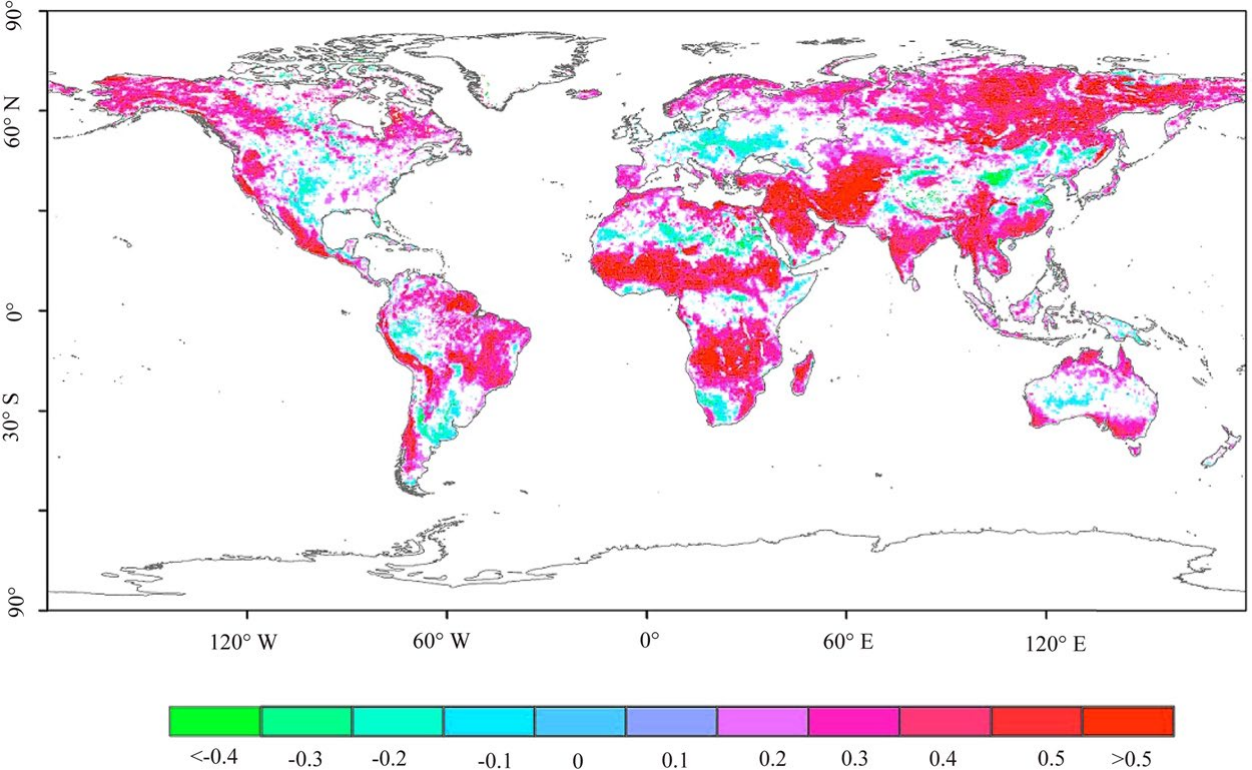
The correlation between monthly soil moisture and next-month precipitation (only significant results are presented at p < 0.05) (Created by ArcGIS, version 10.1, http://www.esri.com/).

The spatial variability in hydrological variables (soil moisture, precipitation and evapotranspiration) results in significant complexity regarding SM-P feedback. Global average statistics, although useful, result in overestimation in some regions and underestimation in others. Since SM-P feedback is a local process^27^, we evaluate the feedback in at the pixel scale spatially. The correlation analysis shows that significant SM-P feedback occurs in 47.10% of the total land area (Fig. 3). As expected, a positive correlation dominates the feedback. However, non-negligible negative feedback also exists in some areas. Statistically, 87.09% of the feedback is positive, and 12.91% is negative. Spatially, the positive feedback is mainly located in central and southern Africa and southwestern and northern Asia. By contrast, the hotspots of negative feedback are located in southern South America, northern Africa, western Europe, and eastern Asia.

**Figure 4:**
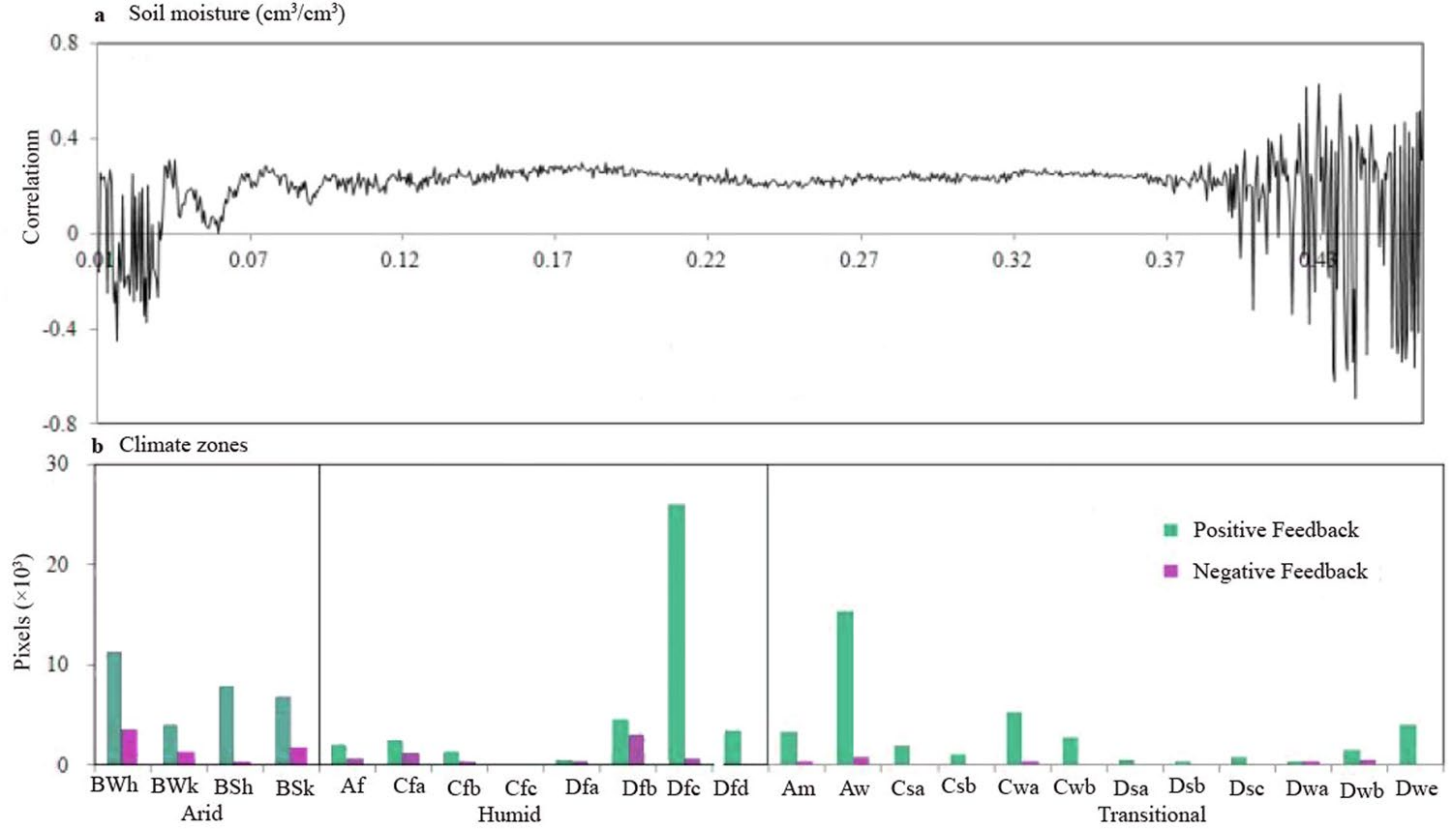
The spatial pattern of SM-P feedback over different soils: (**a**) The average correlation with a soil moisture interval of 0.0005 cm^3^/cm^3^; (**b**) Negative feedback in different climate zones. The first letter (A to D) refers to the broad climate type: tropical, arid, temperate or cold. The second letter (f, m, w/W and s/S) represents the subsequent precipitation conditions. The third letter (**a**–**d**,**h** and **k**) refers to the temperature classification. The definitions of the climate zones can be found in Peel *et al*.^46^. Each pixel represents an area of 0.25 × 0.25 degrees.

SM-P feedback is strongly correlated with the environment and climate^7,14,16,23,24,28^. Thus, we estimate the spatial patterns of feedback in different soils and climate zones. As shown in Fig. 4(a), positive feedback occurs in transitional soils (0.05 < *θ*_*v*_ < 0.40 cm^3^/cm^3^), while negative feedbacks occur in extreme dry (*θ*_*v*_ < 0.05 cm^3^/cm^3^) and wet (*θ*_*v*_ < 0.40 cm^3^/cm^3^) soils. At the climate zone scale (Fig. 4(b)), 27.73%, 37.49% and 34.78% of the positive feedbacks occur in arid, humid and transitional zones, respectively, and cover 45.16%, 43.31% and 65.43% area of each climate zone. The negative feedbacks are mainly located in arid and humid zones, which account for 43.75% and 40.16% of all negative feedback, respectively, and cover 10.73% and 6.99% of the climate zones. Previous studies have provided numerous examples of positive SM-P feedback in transitional zones^14–16,29^. The results of this study demonstrate that negative feedback can occur in dry and wet regions, which could clarify the climate conditions associated with the feedback sign.

**Figure 5:**
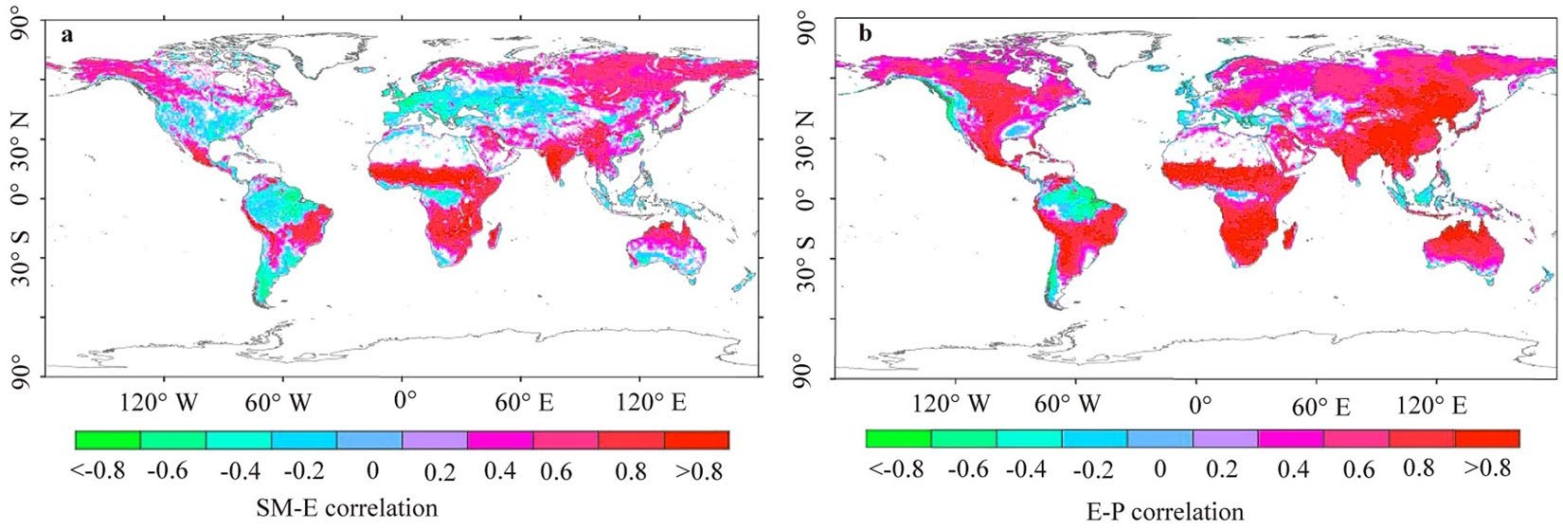
Correlations of (**a**) SM-E and (**b**) E-P (only significant results are presented at p < 0.05) (Created by ArcGIS, version 10.1, http://www.esri.com/).

Koster’s framework is then used to explore the physical mechanism of SM-P feedback. According to the framework, one or three negative correlations of P-E, SM-E and E-P would result in negative SM-P feedback, and positive P-SM correlation has been observed in previous studies^30–34^. It is also upstream of the SM-P feedback loop. Therefore, only the SM-E and E-P correlations are adopted to estimate the physical mechanism of SM-P feedback (Fig. 5). The positive and negative SM-E correlations cover 50.77% and 24.74% of the total land area (Fig. 5(a)). Seneviratne *et al*.^35^ presented probable reasons for the complex SM-E correlation. In transitional climate zones, evapotranspiration exhibits an approximately linear relationship with soil moisture. However, the correlation is weakened in dry soil because of high soil suction (soil moisture limited) and in wet soil because of potential evapotranspiration (energy limited). Spatially, the negative SM-E correlation covers 72.81% area of the negative SM-P feedback, which is concentrated in southern North America, northern South America and western Asia. According to Koster’s framework, the spatial inconsistence between the SM-E and E-P correlations generates the negative SM-P feedback. Since the positive E-P correlation is observed over land (84.38% of the total land area) (Fig. 5(b)), the negative SM-E correlation might be the dominant force associated with negative SM-P feedback.

### Negative SM-P feedback in dry and wet regions

**Figure 6:**
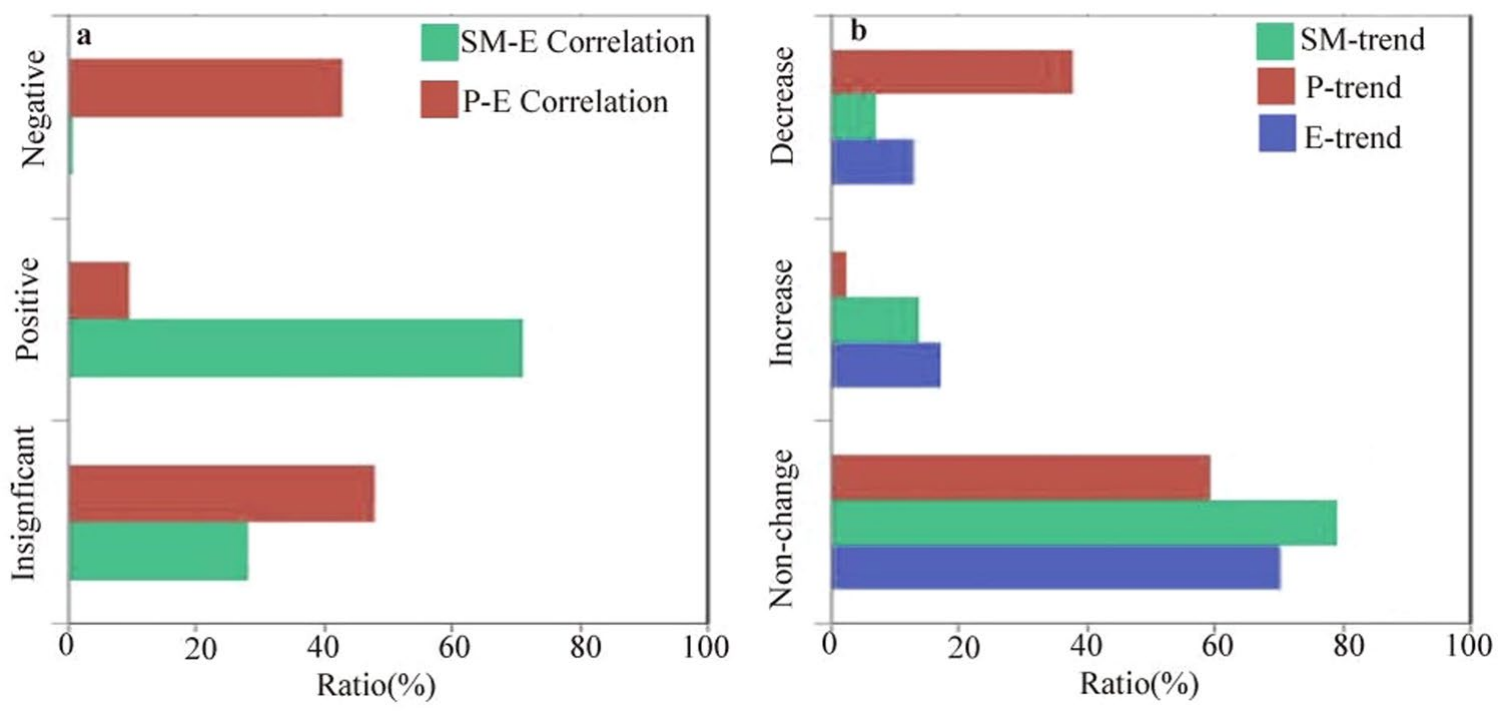
(**a**) Correlations of SM-E and E-P and (**b**) the water change in dry regions with strong negative SM-P feedback. The *y*-axis is the area ratio, which sums to 1.

Positive SM-P feedback has been widely observed in past studies, while negative feedback has been given less attention. Thus, we further investigate the physical mechanism of negative feedback in dry and wet regions and estimate the SM-E and E-P correlations in areas with negative SM-P feedback. As shown in Fig. 6(a), the negative SM-E correlation accounts for approximately half (42.77%) of the negative SM-P feedback in dry regions, while the positive SM-E correlation only accounts for 9.33% of the feedback area. However, the negative E-P correlation occurs in only 0.75% of the negative SM-P feedback area, while the positive E-P correlation accounts for 71.01% of the area. This result further confirms that the negative SM-E correlation accounts for the negative SM-P feedback. We further examine the water change in the areas of negative SM-P feedback in dry regions (Fig. 6(b)). We found that soil moisture decreases in 37.98% of the feedback area and increases in only 2.66% of the area. However, evapotranspiration changes in a relatively small area, represented by a decrease in 12.81% and increase in 17.08% of the feedback area. This finding demonstrates that the negative SM-E correlation in the dry region mainly causes the decrease in soil moisture. This is consistent with the results of a previous study, which found that global soil moisture change adheres to the “drier in dry” paradigm^20^. The minor change in evapotranspiration might be attributed to the soil moisture-limited conditions in the dry region^11^. The statistics show that the relative changes in soil moisture and evapotranspiration are 0.54% and 0.18%, respectively, in the negative SM-P feedback area in the dry region. Thus, the negative SM-P feedback is ultimately observed due to the combination of negative SM-E and positive E-P correlations.

**Figure 7:**
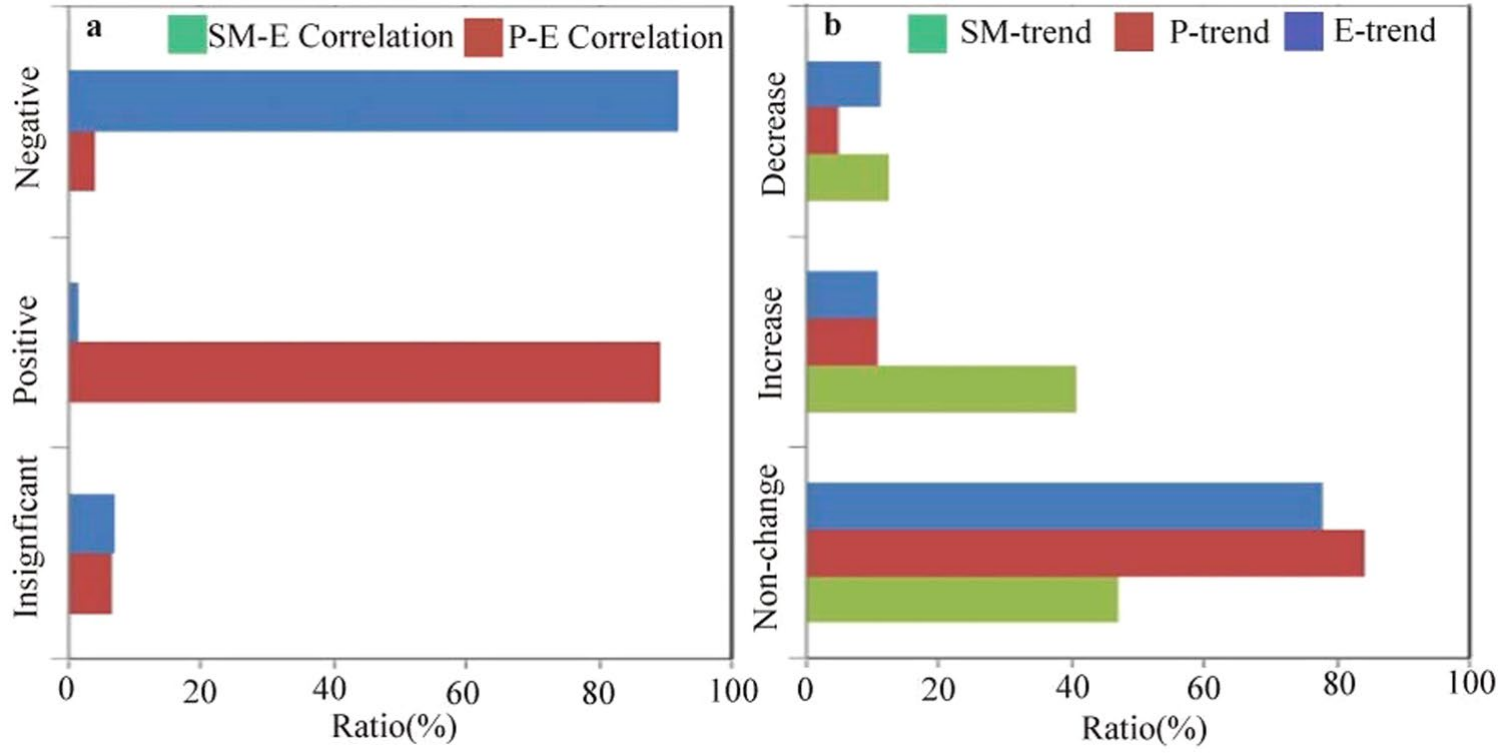
(**a**) Correlations of SM-E and E-P and (**b**) the water change in wet regions with strong negative SM-P feedback. The *y*-axis is the area ratio, which sums to 1.

The negative feedback over humid zones implies that limited precipitation occurs over these wet regions. Wei *et al*.^36^ attributed the feedback to the combined effects of the temporal autocorrelation of precipitation and the soil moisture memory. However, it is difficult to understand the spatial heterogeneity of the feedback. Thus, we investigate this mechanism based on Koster’s framework. As shown in Fig. 7(a), the negative SM-E and positive E-P correlations cover 91.65% and 89.27% of the negative SM-P feedback area in wet regions, respectively. This result indicates that the SM-E correlation plays a more important role in negative SM-P feedback in wet regions than it does in dry regions. Figure 7(b) presents the water changes in the negative SM-P feedback area in wet regions. The soil moisture decreases insignificantly, decreasing in 11.38% of the feedback area and increasing in 10.97% of the area. However, evapotranspiration increases over a larger area (40.64% of the feedback area) but only decreases in 12.37% of the area. This finding indicates that the increasing evapotranspiration dominates the negative SM-E correlation in wet regions, potentially because of the variability in the near-surface temperature. It was previously reported that 80% of the overall near-surface temperature is associated with soil moisture change^37^. The near-surface temperature is 9.67 °C in the negative SM-P feedback area and −1.70 °C in the positive feedback area. According to Seneviratne^11^, the evapotranspiration change is energy limited in wet regions. Therefore, the high temperature tends to enhance the vapor pressure deficit and evaporative demand, which increases evapotranspiration. The increased evapotranspiration further enhances precipitation, which results in negative SM-P feedback under the condition of increasing soil moisture.

## Discussion

SM-P feedback is crucial for climate studies. Positive feedback has been widely noted in previous studies, while negative feedback has been given less attention. In this study, we identified the sign of the feedback based on satellite remote sensing and ground observation data sets. Then, we investigated the physical mechanism of the feedback through Koster’s framework. Methodologically, the feedback is represented by the correlation between monthly soil moisture and next-month precipitation. Although the index is simple, the correlation coefficient (r) helps to capture the causal relationship. Through Koster’s framework, the physical mechanism of SM-P feedback was identified based on the interactions between soil moisture, evapotranspiration and precipitation.

Our results demonstrate that both positive and negative SM-P feedbacks occur simultaneously over land. Specifically, positive feedback mainly occurs in transitional regions, while negative feedback occurs in extreme dry and wet regions. Negative SM-P feedback mainly depends on the SM-E correlation. We further reveal that negative SM-E correlation is mainly attributed to decreasing soil moisture in arid regions and increasing evapotranspiration in wet regions. Our results have profound meaning for global water research. For example, the negative SM-P feedback in humid regions reflects the increased risk of drought in wet areas, which has been confirmed in several previous studies^17,38^. The hotspots presented in our study allow researchers and decision makers to devote more attention to high-risk regions.

Our results demonstrate that the SM-E correlation is the dominant factor for the sign of SM-P feedback. Therefore, comprehending the variability in the SM-E correlation is critical for understanding the physical mechanism of SM-P feedback. However, the effect of soil moisture on evapotranspiration varies with the environment and climate, leading to significant uncertainty regarding evapotranspiration estimation^39^. Therefore, the response of the SM-P correlation to global change should be fully investigated. Climate change has intensified the global water cycle in recent decades^40–44^, which has unavoidably altered SM-P feedback. Thus, the relationship between the water cycle and SM-P feedback must be taken into consideration in future studies.

## Methods and Materials

### Methods

Most studies have interpreted the feedback based on the energy balance^9,14,16,17,22,23^. In this way, the effects of soil moisture on the latent and sensible heat fluxes, convective system and boundary layer are investigated using land-atmosphere models. Although the method addresses land surface-atmosphere interactions, it contains uncertainty because of the complex interactions of the variables, weakening the reliability of the results^26^. Therefore, we investigate the feedback based on a statistical analysis. The coefficient of correlation (*r*) is adopted to identify the sign of SM-P feedback^15,36^. Considering the time lag effect of soil moisture on precipitation, we evaluate the correlation between monthly soil moisture and precipitation in the next month^15,45^.

**Figure 8:**
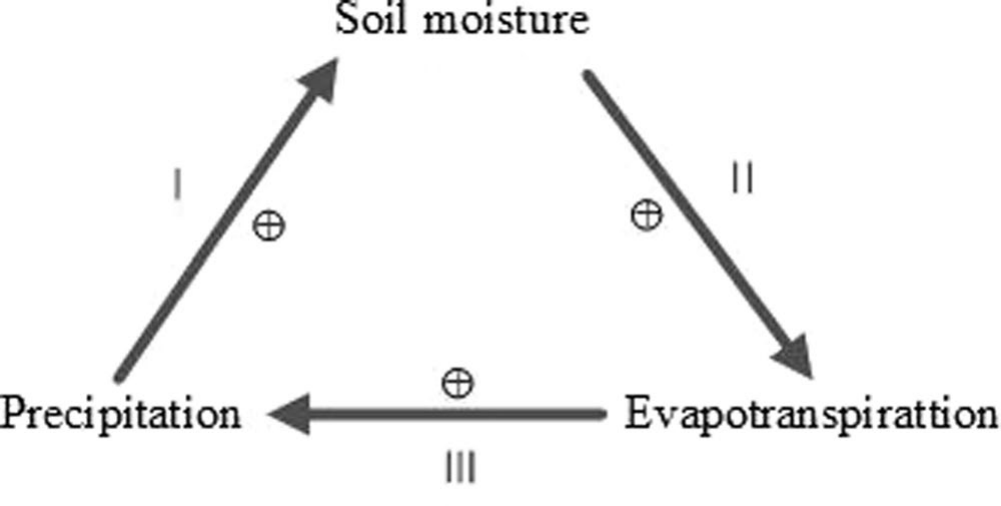
SM-P feedback loop.

It is difficult to evaluate the physical mechanism of SM-P interaction using soil moisture and precipitation information because the latter variable has a direct impact on the former, and their causal relationship cannot be easily identified^46^. Evapotranspiration links the surface water and energy balances, which affect the feedback^32,34,47–50^. Therefore, we estimate the feedback by introducing evapotranspiration into the feedback loop. Koster *et al*.^48^ proposed a feedback framework based on the concept of water balance. It sub-divided the feedback as the coupling of the wetting soil by precipitation (P-SM), enhancement evapotranspiration by the wetted soil (SM-E), and enhancement precipitation by the high evapotranspiration (E-P) (Fig. 8). From this framework, the sign of the feedback can be easily identified. According to the feedback loop, negative SM-P feedback occurs when one or three of the sub-divisions is/are negative.

### Materials

The global water cycle has intensified in recent decades. We select data from 1982–2011 to capture global water and climate change. Data sets of satellite remote sensing and ground observations are used to investigate the SM-P feedback in this study. Global soil moisture data from the Climate Change Initiative (CCI) and global precipitation data from the Global Precipitation Climatology Centre (GPCC) are adopted to identify the feedback. The evapotranspiration data from FLUXNET and near-surface temperature from the University of Delaware Air Temperature & Precipitation data set are used to estimate the physical mechanism and the climate conditions associated with the feedback. To analyze the spatial patterns, we evaluate the feedback in different climate zones. The zones are defined using the updated classification of Koppen-Geiger^46,51^ and then classified as humid, arid or transitional zones^14^. The data sets are introduced as follows.

#### Soil moisture data

Satellite remote sensing provides global soil moisture information over a large spatial view. CCI provides the longest time series of global satellite soil moisture from 1979–2013, which is used in this study. The data are combined by active and passive microwave satellite observations. The active data sets are generated by the University of Vienna and are based on observations from the C-band scatterometer of the European Remote Sensing satellites (ERS-1 and ERS-2) and the Meteorological Operational Satellite (MetOp-A). The passive data sets are based on observations from the Scanning Multichannel Microwave Radiometer (SMMR), the Special Sensor Microwave/Imager (SSM/I), the Tropical Rainfall Measuring Mission microwave imager (TRMM TMI) and the Advanced Microwave Scanning Radiometer-Earth Observing System (AMSR-E). The accuracy of the CCI data is acceptable when validated using global ground-based observations, and the mean correlation coefficient (*r*) and root mean square error (RMSE) are 0.46 and 0.04 cm^3^/cm^−3^, respectively^52^.

#### Precipitation data

GPCC precipitation data are used in this study and were obtained from the Deutscher Wetterdienst of Germany’s National Meteorological Service. The data include precipitation climatology for global land areas based on objective analyses of climatological norms, and the database includes information from approximately 67,200 rain gauge stations^35^. The relative sampling error of gridded monthly precipitation ranges from ± 7–40% of the true mean. The Full Data Reanalysis V.7 (1901–2013) data set is adopted in this study. The spatial resolution was resampled to 25 km to match the soil moisture data used in this study.

#### Evapotranspiration data

Evapotranspiration data are adopted to investigate the mechanism of SM-P feedback. The data are calculated from FLUXNET eddy covariance-based latent heat and then upscaled to the global scale^53,54^. Validation results showed that the RMSE of latent heat is approximately 0.7*MJ*/*m*^2^/*day*, and the correlation coefficient *r* is approximately 0.9. The data were obtained from (https://www.bgc-jena.mpg.de/geodb/projects/FileDetails.php). We then transform the latent heat (*LE*) to evapotranspiration (*ET*) by the relationship $LE=\lambda \cdot ET$ (where $\lambda =2.45MJ/kg$ is the latent heat of vaporization).

#### Climate data

Global surface temperature data are used to understand the climate condition of the SM-P feedback. The University of Delaware Air Temperature & Precipitation data are used in this study (http://www.esrl.noaa.gov/psd/data/gridded/). To investigate the spatial pattern of the feedback, the climate zones are classified based on the work of Koppen-Geiger. The classification defines the zones based on a combination of precipitation and air temperature. Peel *et al*.^46^ updated the classification based on long-term monthly data sets of precipitation and temperature. Polar regions cover large areas of ice and snow, which are excluded from our study. Meanwhile, we re-classify arid regions as defined by Peel *et al*.^46^. Humid regions are zones without a dry season, and transitional regions are the final type of climate zone^20^. The three climate regions (arid, humid and transitional) cover 30.39%, 42.83% and 26.78% of the global land area, respectively.
